# Genetic Variability in DNA Repair Proteins in Age-Related Macular Degeneration

**DOI:** 10.3390/ijms131013378

**Published:** 2012-10-18

**Authors:** Janusz Blasiak, Ewelina Synowiec, Antero Salminen, Kai Kaarniranta

**Affiliations:** 1 University of Lodz, Faculty of Biology and Environmental Protection, Department of Molecular Genetics, Pomorska 141/143, Lodz 90-236, Poland; E-Mail: ewelinas@biol.uni.lodz.pl; 2 Department of Neurology, Institute of Clinical Medicine, University of Eastern Finland, Kuopio FI-70211, Finland; E-Mail: antero.salminen@uef.fi; 3 Department of Neurology, Kuopio University Hospital, Kuopio FI-70211, Finland; 4 Department of Ophthalmology, Institute of Clinical Medicine, University of Eastern Finland, Kuopio FI-70211, Finland; E-Mail: Kai.Kaarniranta@kuh.fi; 5 Department of Ophthalmology, Kuopio University Hospital, Kuopio FI-70211, Finland

**Keywords:** age-related macular degeneration, AMD, DNA repair, genetic polymorphism

## Abstract

The pathogenesis of age-related macular degeneration (AMD) is complex and involves interactions between environmental and genetic factors, with oxidative stress playing an important role inducing damage in biomolecules, including DNA. Therefore, genetic variability in the components of DNA repair systems may influence the ability of the cell to cope with oxidative stress and in this way contribute to the pathogenesis of AMD. However, few reports have been published on this subject so far. We demonstrated that the c.977C>G polymorphism (rs1052133) in the *hOGG1* gene and the c.972G>C polymorphism (rs3219489) in the *MUTYH* gene, the products of which play important roles in the repair of oxidatively damaged DNA, might be associated with the risk of AMD. Oxidative stress may promote misincorporation of uracil into DNA, where it is targeted by several DNA glycosylases. We observed that the g.4235T>C (rs2337395) and c.–32A>G (rs3087404) polymorphisms in two genes encoding such glycosylases, *UNG* and *SMUG1*, respectively, could be associated with the occurrence of AMD. Polymorphisms in some other DNA repair genes, including *XPD* (*ERCC2*), *XRCC1* and *ERCC6* (*CSB*) have also been reported to be associated with AMD. These data confirm the importance of the cellular reaction to DNA damage, and this may be influenced by variability in DNA repair genes, in AMD pathogenesis.

## 1. Introduction

Age-related macular degeneration (AMD) is an eye disease affecting mainly the elderly; it is the main cause of blindness in the developed countries. AMD is characterized by a degeneration of the retinal pigment epithelium and photoreceptors (rods and cones) and a thickening of the Bruch’s membrane in the macula. In general, AMD is divided into dry (atrophic) and wet (exudative) AMD forms. The protrusion of choroidal neovascularization into the retinal layers is a diagnostic sign of wet AMD. The pathogenesis of AMD is complex and involves interactions between multiple environmental, life-style and genetic factors [1]. However, almost all of the environmental and life-style AMD risk factors are associated with oxidative stress. Since most of these genetic factors are related to oxidative stress, they seem to be of special significance in the pathology of AMD. The cellular reaction to oxidative stress is determined by at least three elements: the presence of small antioxidants, including some vitamins and glutathione, the activity of antioxidant enzymes, e.g., superoxide dismutase, catalase and the efficacy of DNA repair. The last factor depends on the expression of genes encoding for DNA repair proteins, this being mainly regulated by the interaction of regulatory proteins and specific sequences in the DNA repair genes. Therefore, this interaction may be affected by sequence changes in these genes, especially when they are located in the coding or regulatory regions of the genes. It should be noted that important regulatory elements might be placed not only in the promoter, but anywhere in the gene’s base sequence. Such changes in the sequence of the gene may occur in a considerable part of a population and then they are called polymorphisms. Genetic polymorphisms are not pathogenic *per se*, but they may contribute to a pathological phenotype in conjunction with some environmental conditions. If interaction between a transcription factor and its target gene sequence is disturbed by a change in this sequence, this may result in changes in the final protein product of this gene and the initiation of pathological events [2].

AMD has been associated with variations in several genes encoding products important for pathological processes linked to AMD-inflammation, oxidative stress, and angiogenesis [3]. These include genes encoding complement factors H, B and I, complement component 2, high temperature required factor A-1, toll-like receptor 4, vascular endothelial growth factor, apolipoprotein E, fibulin 5 and 6, ATP-binding transporter protein and age-related maculopathy susceptibility 2 locus [4]. The genetic predisposition to a disease is determined by the interaction of high-risk variants with low- and medium-penetrance genes [5]. Many studies have revealed an association between a polymorphism in the DNA repair genes and diseases, mostly cancer [6–8]. However, very few studies have been conducted on the role of genetic variability in DNA repair genes in the pathogenesis of AMD. This is somewhat surprising given the established role of oxidative stress in this disease process. This review will discuss the published results and ongoing projects on the association, or the lack of any association, between certain polymorphisms in the DNA repair genes and the occurrence and progression of AMD.

## 2. XRCC1

### 2.1. The Protein and the Gene

The XRCC1 (X-ray repair complementary group 1) protein plays a fundamental role in DNA base excision repair (BER), but it may also be involved in non-homologous end joining and the single-strand break repair pathway [9,10] (Table 1). In BER, a damaged base is recognized and excised, resulting in an AP (apurinic or apyrimidinic) site, which is then nicked with the resulting single-stranded termini being processed if necessary, *i.e.*, the gap is filled and the missing phosphodiester bond is recovered. XRCC1 is involved in all these steps [10]. It is a scaffold protein, which is functionally and physically associated with many BER proteins, facilitating their enzymatic function, although it lacks any enzymatic activity itself (Table 1).

The human *XRCC1* gene spans about 33 kilobases in the chromosomal region 19q13.2 (Table 1). It has 17 exons and its product, the XRCC protein, contains 633 amino acids with a molecular weight of almost 7 kDa.

More than 700 single nucleotide polymorphisms (SNPs) in the *XRCC1* gene have been registered in the SNP database (http://www.ncbi.nlm.nih.gov/SNP). However, only three of them have been extensively studied: p.R280H (a change from arginine to histidine at Codon 280, g.44056412G>A, rs25489), p.R194W (a change from arginine to tryptophan at Codon 194, g.44057574A>G, rs1799782) and p.R399Q (a change from arginine to glutamine at Codon 399, Arg399Gln, g.44055726T>C, rs25487). These polymorphisms have been associated with several pathologies, including gastric [19], colorectal [20], skin [21], breast and other cancers [22]. Polymorphic variants of the *XRCC1* gene have been reported to be associated with inflammation-related malignancies [23–26].

### 2.2. XRCC1 in AMD

As mentioned above, oxidative stress is one of the most important factors in the pathogenesis of AMD. The stress is associated with increased levels of damage to cellular molecules, including DNA [27]. Oxidative stress-resulted DNA damage mainly takes the form of DNA base modifications, which are targeted by BER. Therefore, proper functioning of this DNA repair system may be important in the pathogenesis of AMD. Such functioning requires proper sequences of genes, the products of which are important for this system and every departure from these sequences may result in the deregulation of BER and may have serious phenotypic consequences.

Since XRCC1 is one of the most important BER proteins, polymorphism of its gene can play a role in AMD pathogenesis, but only one study has investigated this phenomenon [28]. That study enrolled 120 AMD patients and 205 controls and employed RLFP-PCR to genotype the two rs1799782 (c.580C>T, p.R194W) and rs25487 (c. 1196A>G, p.Q399W) sense polymorphisms of the *XRCC1* gene. No significant difference was found in the distributions of genotypes and alleles of these polymorphisms between AMD patients and controls. Stratification of the patients by AMD subtype (dry/wet) failed to also detect any association between these polymorphisms and AMD. The authors also investigated polymorphisms of the *XPD* gene, but they did not perform an analysis of combined genotypes of the *XRCC1* and *XPD* genes.

## 3. XPD (ERCC2)

### 3.1. The Protein and the Gene

The XPD (the *Xeroderma pigmentosum* group D) protein is a subunit of the transcription factor TFIIH, which plays a crucial role in the transcription governed by RNA polymerase II, and it is the coupling factor between transcription and nucleotide excision repair (NER) of DNA (Table 1). It has 760 amino acids and a molecular weight of about 87 kDa. XPD is an ATP-dependent helicase unwinding DNA in the 5′–3′ direction. As its name suggests, a defective version of XPD may be associated with *xeroderma pigmentosum* (XP), a rare and severe recessive disease resulting in abnormal sensitivity to UV radiation and the development of skin cancers.

The gene encoding XPD, the *XPD* gene (aka *ERCC2*) is 19,631 bp long, has 22 exons and lies at 19q13.3 (Table 1). Since TFIIH is involved in several important processes, mutations in the *XPD* gene may result in a disease phenotype, but the relationship genotype-phenotype is usually complex [11]. Most mutations in *XPD* result from the alterations in the C-terminal part of the XPD protein. These mutations may be associated with XP, Cockayne syndrome and trichotiodystrophy [11]. The most common XPD mutation is located at R683.

Over 500 SNPs in the *XPD* gene can be found in the SNP databases, with nearly 200 being placed in introns, although some of these SNPs may affect the splicing pattern of primary transcript [29]. The two common polymorphisms, which actually change the amino acid sequence in the XPD gene are c.1021G>A (p.D312N, rs1799793) and c.2329A>C (p.K751Q, rs13181). These polymorphisms have been associated with prostate [30] and bladder cancer [31]. There was no association of p.D312N with head and neck cancer [32]; p.K751Q did not display an association [31]. Furthermore there are negative or conflicting results on the role of these polymorphisms in lung cancer [33], basal cell carcinoma [34–36], breast [37,38], head and neck [39] and colorectal cancer [40].

### 3.2. XPD in AMD

The association between the two polymorphisms of the *XPD* gene, p.D312N and p.K751Q, and AMD has been investigated in one study [28]. The genotype Q/Q of the p.K751Q polymorphism displayed a protective role against the development of AMD. Haplotype analysis confirmed the possible involvement of the Q751Q variant in AMD pathogenesis—The risk of AMD occurrence was strongly reduced in carriers of the 312D-751Q haplotype. Stratification of AMD patients according to the disease subtype (dry/wet) revealed that the Q751Q genotype might have a protective effect against the occurrence of the dry form of AMD. The frequency of the Q751Q in the wet AMD patients did not differ from that in the controls. No other reports of any possible involvement of the variability in the *XPD* gene in AMD pathogenesis have been published.

## 4. ERCC6 (CSB)

### 4.1. The Gene and the Protein

The *ERCC6* (excision repair cross-complementing rodent repair deficiency, complementation group 6) gene is located at 10q11.23 (Entrez Gene cytogenetic band) and has 84,171 bases with 21 exons (Table 1). Since there are alternative polyadenylation forms of the gene, its expression results in two mRNAs of 5 and 7.5 kb. The longer mRNA produces a protein of 1493 amino acids, the CSB protein, and defects in this protein were found to be involved in the Cockayne syndrome (CS) phenotype [12]. About 80% of patients with CS have mutations in the *CSB* gene. CSB contains several conserved ATPase motifs, forming a nucleotide-binding fold, as is typically the case for RNA and DNA helicases [13]. CSB plays an important role in coupling NER to arrested transcription complex in transcription-coupled repair (TC-NER), but the protein is not a damage-recognition factor *per se* [41]. CSB was suggested to be involved in the initiation of TC-NER through recognition of and binding to a blocked RNA pol II complex at the site of DNA damage [42] (Table 1). Alternatively, CSB might help remodeling the RNA pol II complex without evoking its release [43]. In addition to the TC-NER, CSB may be involved in the removal of oxidative DNA damage through BER pathways, as suggested by the accumulation of oxidative DNA damage in CSB-deficient cells after oxidative stress [44] (Table 1). Subsequent studies have clarified the role of CSB in the incision process of oxidative base damage [45].

Over 1500 SNPs in the *ERCC5* gene have been registered in the NCBI database (http://www.ncbi.nlm.nih.gov/snp), and several reports indicate that some of these SNPs possess functional significance. The c.–6530C>G polymorphism of the gene (rs3793784) was associated with the modulation of transcription activity through changes in the binding of the Sp1 transcription factor as well as the risk of lung cancer [46]. The rs4253079 polymorphism, which is an A to C change in intron 4 of the gene, was associated with survival of patients with low-grade anaplastic gliomas [47]. Several polymorphisms in the *ERCC6* gene have been associated with bladder [48], laryngeal [49] and oral [50] cancer.

### 4.2. ERCC6 in AMD

*ERCC6* is the most extensively studied DNA repair-gene in AMD. It has been considered as a candidate gene in AMD pathogenesis due to its role in the aging process, DNA repair and the ocular manifestations resulting from the disruption of this gene [51]. The c.–6530C>G polymorphism in the 5′ flanking region of this gene (rs3793784) was chosen to be studied in AMD on the basis of its location in the transcription factors binding region. The C and G allele displayed a different binding pattern to the nuclear proteins. The C/G genotype of the c.–6530C>G polymorphism increased AMD risk independently and potentiated the effect of the rs380390 polymorphism in the complement factor H (CHF) gene, which is a firmly established AMD risk factor [52]. That study revealed elevated expression of the ERCC6 protein in archival ocular tissue of AMD patients in comparison with that in normal eyes, especially in the carriers of the G allele. The authors concluded that the high expression of the ERCC6 protein predisposed the individuals towards AMD. Based on their results, two general mechanisms for this predisposition can be proposed. ERCC6 may interfere with TC-NER or BER, leading to diminished efficacy in the repair of oxidative DNA damage, resulting in degeneration changes occurring in the macula, although it is somewhat difficult to support this hypothesis in view of the increased level of ERCC6. On the other hand, the elevated ERCC6 expression may affect the complement cascade and in this way play a role in the pathogenesis of AMD. This is supported by the results from the analysis of the combined *ERCC6*-*CHF* genotypes. In summary, the results obtained by the authors point to a possible role of DNA repair in AMD pathogenesis and may even have identified specific target molecules in AMD prevention and/or therapy.

The association between the c.–6530C>G polymorphism of the *ERCC6* gene and AMD was investigated using the individual and combined data from three large AMD case-control studies and a prospective population-based study (The Rotterdam Study) [53]. It was shown that the G allele was associated with a small increase in the risk of late AMD in the Dutch population, but this was not confirmed in two non-European studies. The positive association was only noted in the incident analysis and not in the prevalent analysis. In the Dutch study, nine other polymorphisms of the *ERCC6* gene chosen on the basis of their functional relevance, minor allele frequency > 10%, coverage of the main linkage disequilibrium blocks and tagging of the most common haplotypes were not associated with AMD. The G allele did not lead to an elevated *ERCC6* mRNA level in the retina of early AMD patients compared with healthy old individuals. Instead, the authors found that the expression levels of *ERCC6* were lower in retinal pigment epithelium of early AMD patients in comparison to the controls. This phenomenon cannot be easily explained. The apparent discrepancy between the results obtained in that experiment and the results described in the previous paragraph was explained by the difference in the sample sizes. The overall interpretation of the data by the authors was that the c.–6530C>G polymorphism of the *ERCC6* gene had only a minor if any role in the pathogenesis of AMD. Their conclusion is supported by one study in which a genome-wide association scan for AMD was performed in a large sample of cases and controls with no association were found for the c.–6530C>G polymorphism [54].

In addition to SNPs, copy number variations (CNVs) have also been recognized as a source of phenotypic variation in the human population, and these have been investigated in AMD candidate genes. CNVs include deletions, duplications and tandem repeats of segments of genomic DNA that are at least 1 kbase long [55]. CNVs have been linked to several human diseases [56]. No changes in the *ERCC6* gene copy number was observed in wet AMD patients compared to their age-matched controls [57].

## 5. hOGG1

### 5.1. The Gene and the Protein

The human *OGG1* gene (8-oxoguanine DNA glycosylase 1, gene ID 4968, also known as *hMMH*, *MUTM* and *OGH1*) is located at 3p26.2, a locus showing a frequent loss of heterozygosity (LOH) in many types of human cancers, including lung and kidney tumors [14] (Table 1). Alternative splicing of the C-terminal region of this gene gives α-OGG1 (1345 amino acids) and β-OGG1 (2424 amino acids) isoforms of the final product—depending on the last exon of the sequence. The Type 1 alternative splice variant ends with Exon 7 and Type 2 with Exon 8. An alternative exon is located approximately 9 kb downstream [14]. Both variants share the N-terminal region, containing a mitochondrial targeting signal (MTS), essential for their mitochondrial localization. β-OGG1 is transferred to the mitochondria, while α-OGG1 is located in the nucleus since there is a nuclear localization signal (NLS) in Exon 7 [15,58]. The *hOGG1* gene codes for 8-oxoguanine DNA glycosylase 1, removing oxidized DNA bases, including 8-oxoguanine (8-oxoG). It possesses AP (apurinic or apyrimidinic) lyase activity and can cleave the phosphodiester chain (Table 1). The hOGG1 protein initiates the base excision repair (BER) pathway by recognizing and excising the modified base by hydrolyzing the N-glycosidic bond. 8-OH-G is one of the major pre-mutagenic derivatives of the DNA bases, resulting from the exposure to reactive oxygen species (ROS), and it has been used as a biomarker for cellular oxidative stress [59]. 8-OH-G can pair with adenine in double-stranded DNA during the DNA replication leading to a G:C to T:A transversion.

*hOGG1* is highly polymorphic, at least 425 SNPs of this gene have been registered (http://www.ncbi.nlm.nih.gov/snp). Among them, the c.977C>G polymorphism (rs1052133) is the most common polymorphism, which is located at the +977 cDNA position in the α-OGG1-specific Exon 7. This nucleotide variation is associated with an amino acid substitution of serine to cysteine at Codon 326 of the hOGG1 protein (p.S326C) and this may result in functional changes. This polymorphism was shown to reduce the *hOGG1* activity *in vitro*, evidence that the C allele may cause a higher risk of 8-oxoG formation in DNA [60]. The allele frequency of 326C is markedly different among ethnic groups. About 25% of the Japanese population is homozygous at Codon 326 (C/C), but the frequency of this allele seems to be lower in Caucasians [61]. Since c.977C>G is located in the coding region of the *hOGG1* gene it may directly affect protein stability and function. It has been demonstrated that this SNP can be associated with an increased risk of lung, esophagus, prostate and a subset of stomach cancers [59,62–64].

### 5.2. hOGG1 in AMD

In aged rodents, it was demonstrated that the level of *hOGG1* decreased retinal pigment epithelium (RPE) and choroid, leading to a reduced ability to repair oxidative DNA damage [65]. There was no association between the p.S326C polymorphism of the *hOGG1* gene and the occurrence of AMD [66]. In contrast, we demonstrated that the S/C genotype and the C allele significantly increased the risk of AMD in both dry and wet forms [67]. On the other hand, the S/S genotype and the S allele were positively correlated with a decreased risk of this disease and individually for both the dry and wet forms. Although we found an association between the genotypes of this polymorphism and the occurrence of AMD in general, this association seems to be driven by the dry form of the disease. Therefore, if one considers the possibility that this polymorphism may be a useful marker in AMD, it should be focused rather on dry AMD than on AMD in general. It is important in the context of the still unknown mutual relationship between dry and wet forms of AMD. The wet form of AMD may be induced with or without the preceding dry form, therefore markers indicating a preference in developing the dry form are needed in the prognosis of dry-dependent or -independent development of AMD.

## 6. MUTYH

### 6.1. The Gene and the Protein

The human *MUTYH* gene (mutY homolog (*E. coli*) gene ID 4595, also known as MYH; CYP2C) consists of 16 exons spanning a region of 11,147 bp, which encodes a protein of 535 amino acids that displays 41% identity to the *E. coli* MutY [16]. The gene is located on the short arm of the chromosome at 1p34.1 (Table 1). Alternative splicing of this gene produces Type 1 (535 amino acids) and Type 2 (521 amino acids) proteins [16]. The N-terminal region of MUTYH acts as a MTS, and the region near C-terminal has a NLS. The Type 2 protein lacks the first exon containing the MTS and is transferred to the nucleus, while the Type 1 protein is moved to the mitochondria [16]. This gene encodes a DNA glycosylase involved in oxidative DNA damage repair in the BER pathway (Table 1). This enzyme removes an adenine incorporated opposite to 8-oxoG during DNA replication. The MUTYH protein interacts with other BER proteins, such as RPA, APE1, PCNA and MSH6 and its level in the nucleus increases in the S phase compared to the early G1 phase [68,69].

Over two hundred and fifty SNPs of the *MUTYH* gene have been registered in the NCBI SNP database. A G to C transversion located at the +972 cDNA position in the 12^th^ exon of the *MUTYH* gene (the c.972G>C polymorphism, rs3219489) is associated with an amino acid substitution of glutamine to histidine at the 324 codon (p.Q324H) of the MUTYH protein [70]. This polymorphism has been implicated in functional changes in the DNA damage response. A recent study has reported only 64% activity in the SNP-c.972G>C MUTYH enzyme compared with the wild type [71]. This polymorphism may be associated with an increased risk for lung and colorectal cancers in the Japanese population [72–74].

### 6.2. MUTYH in AMD

As far as we are aware, the c.972G>C polymorphism has not been studied in AMD, and we thus investigated the association between the SNP and risk of AMD [67]. We observed some statistically significant associations between the occurrence of AMD and its dry form but due to the borderline nature of these associations, we do not consider them as medically relevant. Further studies will be needed to determine the possible association between AMD and the c.972G>C polymorphism.

## 7. SMUG1

### 7.1. The Gene and the Protein

The human *SMUG1* gene (single-strand-selective monofunctional uracil-DNA glycosylase 1; gene ID 23583, also known as: FDG, UNG3 and HMUDG) is located in the long arm of Chromosome 12 (12q13.11–13.3) [18,75] (Table 1). This gene encodes for a uracil DNA glycosylase (UDG) of the BER pathway that removes uracil, in spite of its name, from single stranded (ssDNA) as well as double stranded DNA (dsDNA) from U:A and U:G pairs and uracil derivates bearing an oxidized group at ring-C5, such as 5-hydroxymethyuracil (5-hmeU), 5-formyluracil (foU) and 5-hydroxyuracil (hmU) [17] (Table 1). 5-hmeU is a result of oxidation of the 5-methyl group of thymine in DNA due to ionizing radiation and other forms of oxidative stress [76]. As SMUG1 removes uracil and 5-hmeU from ssDNA and dsDNA, this enzyme may play a role in the repair of deamination and oxidation damage to DNA. SMUG1 functions better on dsDNA in the presence of the APE1 endonuclease [77]. It was shown that SMUG1 is the major enzyme involved in the removal of 5-hmeU from DNA [77]. SMUG1 is not regulated by the cell cycle and is evenly distributed in the nucleoplasm [78]. Human SMUG1 (hSMUG1) has 270 amino acids (30 kDa) and is to 60% identical and 71% similar to the *Xenopus* enzyme [73]. Over two hundred SNPs in the *SMUG1* gene are now listed in the NCBI base.

### 7.2. SMUG1 in AMD

We analyzed the association between the c.–32A>G polymorphism (rs3087404) of the *SMUG1* gene and the risk of AMD [79]. This SNP is located in the noncoding, regulatory regions of the gene and can affect mRNA stability and degradation and gene expression, and may result in changes in the level or the activity of the encoded enzyme, which can lead to reduced protection against DNA damage. Our findings showed that the G/G genotype of this polymorphism decreased the risk of AMD progression from its dry to wet form, while the A allele increased this risk.

## 8. UNG

### 8.1. The Gene and the Protein

The human *UNG* gene (UNG uracil-DNA glycosylase, gene ID 7374, also known as DGU, UDG, UNG1, UNG2, HIGM4, HIGM5 and UNG15) consists of 7 exons, spans approximately 13.8 kb and is located at 12q23–24.1 [73] (Table 1). It contains differentially expressed TATA-less promoters [80]. The possibility for alternative promoters and splicing of this gene leads to two different isoforms of the major human UNGs: the mitochondrial UNG1 and the nuclear UNG2. UNG1 and UNG2 have a common catalytic domain, and unique *N*-terminal sequences, which are fundamental for correct subcellular targeting [81]. UNGs prefer ssDNA as the substrate, but also effectively excise uracil from dsDNA [82]. The UNG1 concentration is higher in non-proliferating cells, especially in cells rich in mitochondria such as skeletal muscle, liver and heart, while the highest expression of nuclear UNG2 mRNA is observed in proliferating tissues such as testis, colon and the small intestine [83]. According to the NCBI database, 365 SNPs of the UNG gene are known.

### 8.2. UNG in AMD

No previous studies have investigated the genetic polymorphism of the *UNG* gene in AMD. At present, little is known about the functional effects of uracil-processing gene polymorphisms. It has been proposed that they can lead to altered enzyme activity, changing uracil concentrations, increasing uracil misincorporation and in this way, contributing to human diseases [81,83]. We selected the g.4235T>C (rs2337395) polymorphism located in the noncoding region of this gene to study the role of variability in the *UNG* gene in AMD pathogenesis. The results obtained indicated that the dry AMD occurrence was positively correlated with the presence of the C/C genotype and C allele, while the T/T genotype and T allele showed a negative association [79]. No such correlation has been found for wet AMD and the entire group of patients with AMD without distinguishing of dry and wet form, suggesting that this polymorphism may be a specific risk marker for the dry form of AMD. We also noted that the C/C genotype was associated with exerting a protective effect against the progression of the disease from its dry to wet form, and the T allele increased the risk of the progression.

## 9. Discussion

The important role of oxidative stress in AMD pathophysiology and the special role of DNA repair in the response of the cell towards stress are the reasons for focusing on the association between polymorphism of DNA repair genes and the risk of AMD. Recognizing associations between polymorphism of DNA repair genes and AMD occurrence may make it possible to identify those individuals with an increased risk of AMD and to moderate this through some kind of personalized therapy, which should be planned after determining the genes involved. The main problem encountered in investigating the association between genetic polymorphisms and a disease is to recognize/establish the functional significance of these polymorphisms. Polymorphism in DNA repair genes may result in changes in the efficacy of removal of damaged DNA, but these changes may not be easily detected due to complex interactions between the proteins in the DNA repair machinery.

We mentioned above that the only study focusing on the association between polymorphisms of the *XRCC1* gene and AMD did not detect a positive result. The product of the gene, the XRCC1 protein, is essential in BER and in the repair of DNA single strand breaks, although it does not itself possess any enzymatic activity. The effect of polymorphisms of the *XRCC1* gene on the efficacy of DNA repair has been assessed in several studies [84]. The results of these studies are not identical and although some of them have detected an influence of the *XRCC1* genotype on the DNA repair capacity, this influence appeared to be moderate, and some studies have found no effect [85,86]. The influence is expressed by a decreased repair capacity in the cells with the W/W genotype of the Q399W polymorphism [87–89]. These results encourage the search for a clarification of the role of this variant in pathology. Therefore, studies on the function of the polymorphism of the *XRCC1* gene in AMD pathogenesis should be continued.

As we cited above, it has been shown that the Q/Q genotype of the p.K751Q polymorphism of the *XPD* gene could protect against the development of AMD. However, there is no convincing data on any functional effect of this polymorphism on DNA repair, although some reports have described attempts to compare the efficacy of DNA repair in cells obtained from subjects carrying various genotypes of this polymorphism. In fact, there is one report of a marginally lower DNA repair efficacy in cells with the Q/Q genotype [90]. However, other studies with the same assay made on larger samples could not confirm those results [91,92]. The p.K751Q polymorphism of the *XPD* gene is a sense mutation causing an amino acid change within the XPD protein. In general, these kinds of changes may not directly influence the activity of a protein, but instead alter its stability or the stability of some complex that it forms. Results obtained from experiments on XP individuals led to the general conclusion that lower than regular levels of a DNA repair protein are not inevitably detrimental [22]. These results suggest that the observed association between a polymorphic variant of the *XPD* gene and AMD may not necessarily be accompanied by changes in the DNA repair efficacy in the retina.

As stated, the *ERCC6* gene is the DNA repair gene most frequently studied in AMD. However, there are some contradictory results, but research should be continued, especially attempts to define which polymorphisms in that gene may have functional significance as expressed by changes in DNA repair efficacy [93].

There are reports on functional polymorphisms in the *hOGG1* gene, leading to a change in DNA repair capacity. The G allele of the p.R46G polymorphism of the *hOGG1* gene was reported to have a reduced activity for excision of 8-hydroxyguanine (8-oxoG), while the H allele of the W154H polymorphism of this gene was less effective in DNA repair than the S allele of the S326C polymorphism of the gene [94]. Furthermore, the p.W229G polymorphism of the gene was associated with a change in the hOGG1 protein function, resulting in an increased level of genomic 8-oxo-G and hypersensitivity to 8-hydroxydeoxyguanosine and ionizing radiation [95]. Therefore, polymorphisms of the *hOGG1* gene may have functional significance and evoke changes in DNA repair capacity. This, along with our results, may stimulate further studies on the role of genetic variability of the *hOGG1* gene in the pathogenesis of AMD. Due to a complementary function of the *MUTYH* gene relative to *hOGG1*, the role of its variability in AMD should be studied concurrently.

The results we obtained point to the next candidates among the DNA repair genes to play a role in AMD pathogenesis—two genes encoding DNA uracil glycosylases, *UNG* and *SMUG1.* The results of several experiments indicate that uracil may play a role in the pathogenesis of AMD. Firstly, as indicated by recent reports, uracil may be generated in significant amounts in DNA after oxidative stress and thus it may have a major role in the development of AMD [96–98]. Secondly, the presence of uracil in DNA occurs as a result of a deficiency of vitamins, such as folic acid, B12 and B6. A disturbance in the homeostasis of the deoxyribonucleotide pool, an equilibrium that is directly dependent on the adequate availability of certain members of the B-vitamin family, may influence the integrity of DNA [99]. Folate, as 5,10 methylene THF, donates a methyl group to uracil, converting it to thymine, which is used for DNA synthesis and repair. In fact, folate deficiency leads to a low 5,10 ME-THF concentration and in that way may increase the level of uracil incorporation into DNA [100,101]. One mechanism for chromosome breaks is now recognized as a deficient methylation of uracil to thymine, and the subsequent incorporation of uracil into DNA at a rate of four million molecules per cell [100]. Similarly, vitamins B6 and B12 appear to play an important role in maintaining the genomic stability, as well as in recycling folate into the co-enzymatic form used for thymidine synthesis. When there is a deficiency of vitamin B12 tetrahydrofolate is trapped as methyl-THF and the methylene-THF pool, which is required for methylation of dUMP to dTMP, is consequently diminished. Therefore, vitamin B12 deficiency, like folate, should cause uracil to accumulate in DNA [102]. On the other hand, vitamin B6 deficiency causes a decrease in the activity of serine hydroxymethyl transferase, which supplies the methylene group for methylene-THF. If the methylene-THF pool becomes diminished in B6 deficiency, then uracil incorporation would be expected to occur [103]. This misincorporation of dUMP instead of dTMP results in U:A mispairs. Uracil in DNA also can occur as a result of spontaneous cytosine deamination, leading to U:G mispairs. Cytosine deamination has been estimated to take place at a rate of 60–500 events per human genome per day [73,104]. It was demonstrated that tobacco smoking, which is one of the strongest AMD risk factors, was associated with a diminished folate status [105]. Interestingly, the only protective factors for AMD known at present are dietary nutrients [106]. The results from the Women’s Antioxidant and Folic Acid Cardiovascular Study (WAFACS) indicated that daily supplementation with folic acid/B6/B12 over seven years could reduce the risk of AMD in women at increased risk of vascular disease [107].

In summary, there are few investigations into the association between variability in DNA repair genes and AMD, although there is a clear rationale to conduct such studies. It seems that future work should focus on the relationship between AMD and genetic variability in the proteins which are primarily responsible for recognizing and removing oxidative DNA damage, as reflected mainly in the form of oxidatively modified DNA bases. These would include hOGG1, NEIL1/2 and MUTYH as well as other proteins playing an important role in these processes, such as APE1, PARP1 and DNA polymerase β.

## 10. Conclusions

Since oxidative stress is a well-recognized factor in AMD, much research effort should be focused on clarifying the mechanism underlying this relationship. As DNA damage is one of the most serious consequences of stress, variability in DNA repair genes may contribute to this mechanism. However, in spite of this clear rationale, very few studies have attempted to link AMD with polymorphisms in the DNA repair genes and the results of these studies have been sometimes ambiguous. Therefore, further research on this subject is needed.

## Figures and Tables

**Table 1:** DNA repair genes considered in this review, their chromosomal location and protein function.

Gene	Chromosomal location	Protein	Reference
*XRCC1*	19q13.2	Base excision repair Non-homologous end joining Scaffold protein Lack of enzymatic activity	[9,10]

*XPD*	19q13.3	Nucleotide excision repair Transcription-coupled repair TFIIH subunit, DNA helicase	[11]

*ERCC6*	10q11.23	Transcription-coupled repair Base excision repair	[12,13]

*hOGG1*	3p26.2	Base excision repair	[14,15]

*MUTYH*	1p34.1	Base excision repair Removes A opposite to 8-oxoG	[16]

*SMUG1*	12q13.1–q13.3	Removes uracil from DNA	[17]
*UNG*	12q23–24.1	Removes uracil from DNA	[18]
